# Application of Dual-Channel Convolutional Neural Network Algorithm in Semantic Feature Analysis of English Text Big Data

**DOI:** 10.1155/2021/7085412

**Published:** 2021-11-06

**Authors:** Yang Li, Chengbo Yin

**Affiliations:** ^1^International Business School, Qingdao Huanghai University, Qingdao, Shandong 266400, China; ^2^School of Data Science, Qingdao Huanghai University, Qingdao 266427, Shandong, China

## Abstract

The current Internet data explosion is expecting an ever-higher demand for text emotion analysis that greatly facilitates public opinion analysis and trend prediction, among others. Therefore, this paper proposes to use a dual-channel convolutional neural network (DCNN) algorithm to analyze the semantic features of English text big data. Following the analysis of the effect of CNN, artificial neural network (ANN), and recurrent neural network (RNN) on English text data analysis, the more effective long short-term memory (LSTM) and the gated recurrent unit (GRU) neural network (NN) are introduced, and each network is combined with the dual-channel CNN, respectively, and comprehensively analyzed under comparative experiments. Second, the semantic features of English text big data are analyzed through the improved SO-pointwise mutual information (SO-PMI) algorithm. Finally, the ensemble dual-channel CNN model is established. Under the comparative experiment, GRU NN has a better feature detection effect than LSTM NN, but the performance increase from dual-channel CNN to GRU NN + dual-channel CNN is not obvious. Under the comparative analysis of GRU NN + dual-channel CNN model and LSTM NN + dual-channel CNN model, GRU NN + dual-channel CNN model ensures the high accuracy of semantic feature analysis and improves the analysis speed of the model. Further, after the attention mechanism is added to the GRU NN + dual-channel CNN model, the accuracy of semantic feature analysis of the model is improved by nearly 1.3%. Therefore, the ensemble model of GRU NN + dual-channel CNN + attention mechanism is more suitable for semantic feature analysis of English text big data. The results will help the e-commerce platform to analyze the evaluation language and semantic features for the current network English short texts.

## 1. Introduction

Statistics indicate that over half of the global publications are in English, and 80% of the web pages or online information is in English. Meanwhile, various English texts (such as news, comments, and e-mail) are filling all aspects of people's life and work [1]. Therefore, the research on new English text semantic feature extraction and understanding methods can solve such problems in artificial intelligence (AI) as text classification, machine translation (ML), automatic question answering, text generation, and human-computer interaction (HCI) and promote the interlanguage communication [2, 3].

With the technological maturity of natural language processing (NLP) in AI, automatic English text semantics can quickly understand the international situation, grasp the orientation of international opinion, and ensure national information security. Therefore, with the development from natural language processing (NLP) to natural language understanding (NLU), people's attention has shifted to semantic understanding methods and text semantic feature extraction mechanisms [4].

Here, the realization of English semantic feature analysis is mainly studied based on big data of English text using the dual-channel convolutional neural network (CNN) algorithm. Then, the model with the highest accuracy in English semantic feature extraction is found through comparative analysis of different models. Innovatively, the attention mechanism, gated recurrent unit (GRU) neural network (NN), and long short-term memory (LSTM) NN are added to the dual-channel CNN algorithm, thereby greatly improving the accuracy of the model.

## 2. Related Works

In the context of big data, the mechanism to analyze the semantic features of English text using the dual-channel CNN algorithm has been studied by many scholars.

Mamoon et al. found that deep neural network (DNN) had achieved great success in semantic segmentation, but its real-time application was still facing challenges. Due to a multitude of feature channels, parameters, and floating-point operations, the network had a slow speed with huge amounts of computation, which was not desirable for real-time tasks, such as robot and autopilot. However, most methods often sacrificed spatial resolution to achieve real-time reasoning speed, resulting in poor performance [5]. Wang and Xu proposed a feature fusion depth projection CNN, which mainly used a new residual block, stepwise res block, to mine high-level semantic features while retaining low-level details. The framework used a specially designed feature fusion module to further balance the features obtained from different levels of the backbone network [6]. Javed et al. proposed a new generation antagonism network. The experimental analysis showed that loss reconstruction using low-level loss and high-level structural similarity loss was very effective in obtaining visually credible and consistent texture [7]. Fraz et al. constructed a deep network for simultaneous segmentation of microvessels and nerves in conventional staining histological images, which could predetect embedded feature attention blocks and uncertainties [8]. Yong et al. put forward a bidirectional feature pyramid network, which further enhanced the detection and classification of some types of obstacles using the multilevel detail features of the bottom layer and the strong semantic features of the high layer in the network structure. The detection and classification performance of the proposed method was evaluated on the self-built dataset. Ablation experiments and performance tests were carried out on open datasets. Experimental results showed that the algorithm had the best detection performance [9]. Rosewelt and Renjit discovered that there was an abundant number of relevant and irrelevant data in the current Internet resources. Semantic analysis played an important role in text mining. In this case, to extract relevant data successfully, data classification should be combined with semantic-based text summarization. Therefore, a new feature selection algorithm based on semantic analysis was proposed, which could select relevant data of similar index from local repository or World Wide Web (WEB) application [10]. Wang et al. proposed a latent Dirichlet allocation (CL-LDD) topic model combined with big data. The results showed that the CL-LDA model could well adapt to the short text topic mining task in outer hair cells (OHCs) with sparse semantics and very little co-occurrence information. The research results could help OHCs provide accurate information and improve service quality [11]. Lou and Shi recognized and analyzed different images through a series of algorithms, such as image feature value extraction, recognition, and convolution [12]. Razzaghi et al. established a new method to learn the perceptual grouping of features extracted by CNN to represent the image structure. In CNN, the spatial hierarchical relationship between high-level features was not considered. To do so, the perceptual grouping of features was utilized. To consider the intrarelationship between feature maps, an improved guided co-occurrence block was proposed and applied to some known semantic segmentation and image classification datasets, which achieved excellent performance [13]. Yang et al. proposed a spatial synthesis technique to generate meaningful synthetic virtual data for acoustic scene classification. A large number of experiments on synthetic data and real acoustic scene classification data sets show that multiscale semantic feature fusion and label smoothing spatial hybrid data enhancement can improve the acoustic scene classification performance of DNN [14].

The above research of CNN in different fields has promoted the development and maturation of the CNN algorithm, as well as its application to the semantic feature analysis of English text. Meanwhile, the above research results suggest that different types of NN will cause great differences in the accuracy of the data. Therefore, different CNN algorithms will be selectively used here to maximize the English text analysis ability of the model.

## 3. Model Establishment and Scheme Design

### 3.1. Affective Analysis of English Texts

#### 3.1.1. Artificial Neural Network (ANN)

**Figure 1:**
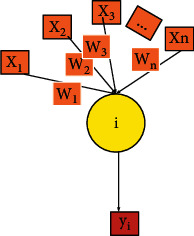
A neuron structure.

ANN is a relatively simple deep learning (DL) model, which can simply imitate the working characteristics of the human brain. A complete ANN is composed of neurons, the structure of which is shown in Figure 1.

In Figure 1, *X*_1_, *X*_2_,…, and *X*_*n*_ represent the input values of input neurons at the input end, and *θ* stands for the bias term. *W*_1_, *W*_2_,…, and *W*_*n*_ denote the weights corresponding to the input values and bias terms. *y*_*i*_ indicates the output of neurons. *i* is a neuronal node, and *y*_*i*_ can be calculated as follows:

(1) $$\begin{array}{c} y_{i}=f\left(\sum_{i=0}^{n}w_{i}x_{i}\right), \end{array}$$

where *f* represents the activation function, in which the sigmoid activation function and hyperbolic tangent activation function are widely used. The expression of the sigmoid activation function is shown in the following equation:

(2) $$\begin{array}{c} f\left(x\right)=\frac{1}{1+\exp\left(-x\right)}. \end{array}$$

**Figure 2:**
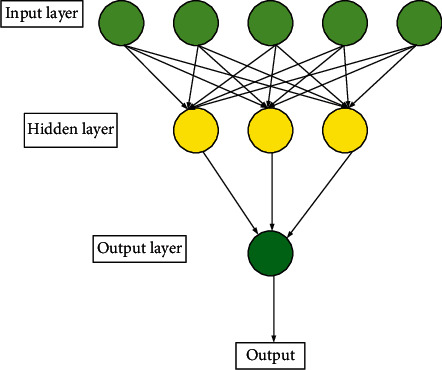
Structure of an ANN.

An ANN is formed through the combination of many neurons.  Figure 2 depicts an ANN with the simplest structure.

The input layer gets the input vector and outputs the calculation result to the hidden layer. Afterward, the hidden layer outputs the calculation results to the output layer, and finally, the output result is obtained in the output layer. Although the ANN seems to be very simple, due to the characteristics of its results, neurons are interconnected with those in the next layer. Therefore, when the network becomes deeper, the parameters surge, the calculation becomes more complex and leads to unsatisfactory results.

#### 3.1.2. CNN

**Figure 3:**
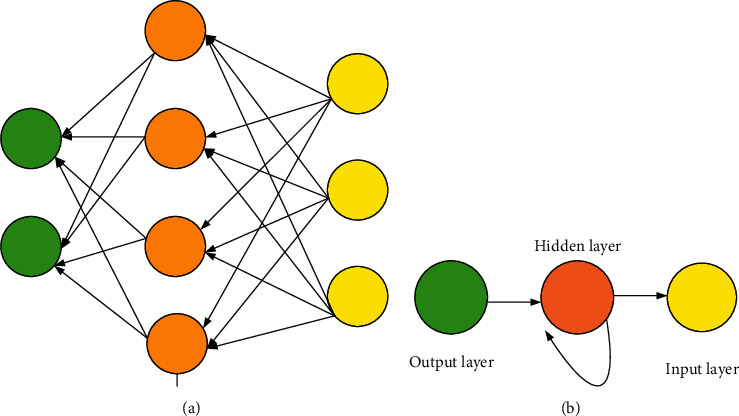
RNN structure. (a) Structure diagram. (b) Simplified structure diagram.

CNN is also a multilayer network structure, which is mainly composed of the convolution layer, pooling layer, and fully connected layer. After the convolution calculation, the convolution layer can select the local features of the upper layer. The essence of photo convolution in image processing is the image filtering process using the convolution kernel. The convolution calculation of the image is shown in the following equation:

(3) $$\begin{array}{c} f\left(x,y\right)*w\left(x,y\right)=\sum_{s=-a}^{a}\sum_{t=-b}^{b}w\left(s,t\right)f\left(x-s,y-t\right), \end{array}$$

where *f*(*x*, *y*) represents the point gray value of the selected image in a coordinate system, *w*(*x*, *y*) denotes the convolution kernel, and *a*, *b* denote the size of the convolution kernel. Individual neurons from different layers are completely interconnected in the fully connected layer of CNN. The fully connected layer can gather the data text features extracted by the previous network layers. Compared with other structures in the CNN model, the fully connected layer contains the most parameters, where many computations in the CNN model are accomplished. Therefore, the model computation can be reduced by introducing the dropout technology in the fully connected layer while improving the generalization ability. Meanwhile, a softmax classifier will be added to the fully connected layer to calculate the probability of a text being a specific emotion and determine the emotional tendency of the text, as shown in Figure 3.

#### 3.1.3. Recurrent Neural Network (RNN)

**Figure 4:**
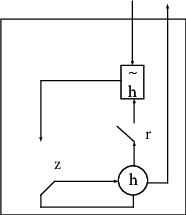
GRU structure.

RNN is a network structure connected into a loop, in which the output of the neural unit relates to the current input, as well as the value of the previous time. This structural feature can deal with the timing problem, as shown in Figure 4.

The calculation of RNN can be expressed as in equations (4) and (5).

(4) $$\begin{array}{c} O_{i}=g\left(V\cdot S_{i}\right), \end{array}$$

(5) $$\begin{array}{c} S_{i}=f\left(U\cdot X_{i}+W\cdot S_{i-1}\right), \end{array}$$

where *X* represents the input data of the model, *U* denotes the weight vector between the hidden layer and the input layer, *S* stands for the data in the hidden layer, *V* indicates the weight vector between the output layer and the hidden layer, *O* refers to the output data of the model, and *W* is the weight between the hidden layer and the hidden layer. However, the oversized training text data cause long-term dependence and over-lengthy sequences that will lead to gradient explosion or disappearance. Given these shortcomings, the application of RNN is greatly reduced.

#### 3.1.4. LSTM NN

The hidden layer of LSTM NN is composed of three gate structures, including the output gate, the forget gate, and the input gate, which can control information transmission in the *U*-form. In each gate, there is a point multiplication operation and a sigmoid layer. The output value range of the sigmoid layer is [0,1], which can describe the amount of information passed by each part, where 0 means no pass and 1 means all pass.

(1) The input layer information *C*_*t*_ is calculated. *W*_*ij*_ represents the weight vector between the input data and the hidden layer, *w*_*jc*_ denotes the output weight of the previous time, and *b*_*c*_ stands for the offset.
  (6) $$\begin{array}{c} C_{t}=\tanh\left(w_{ij}x_{t}+w_{jc}C_{t-1}+b_{c}\right). \end{array}$$
(2) The input gate *i*_*t*_ is calculated. *W*_*xi*_ represents the weight between the input gate and the input information, *w*_*ih*_ denotes the weight between the input gate and the output at the previous time, *w*_*ci*_ stands for the weight between the input gate and the cell at the previous time, and *b*_*i*_ is the offset.
  (7) $$\begin{array}{c} i_{t}=\psi \left(w_{xi}x_{i}+w_{hi}h_{i-1}+w_{ci}c_{t-1}+b_{i}\right). \end{array}$$
(3) Similarly, the forget gate is calculated.
  (8) $$\begin{array}{c} f_{t}=\psi \left(w_{xf}x_{t}+w_{hf}h_{t-1}+w_{cf}c_{t-1}+b_{f}\right). \end{array}$$
(4) The status value in cell is calculated.
  (9) $$\begin{array}{c} c_{t}=f_{t}⊗c_{t-1}+i_{t}⊗c_{t}. \end{array}$$
(5) The status value of the output gate is calculated.
  (10) $$\begin{array}{c} O_{t}=\psi \left(w_{xo}x_{t}+w_{ho}h_{t-1}+w_{co}c_{t-1}+b_{o}\right). \end{array}$$
(6) The final output of the LSTM network is expressed as follows:
  (11) $$\begin{array}{c} h_{t}=o_{t}⊗\;\tanh\left(c_{t}\right). \end{array}$$

#### 3.1.5. GRU NN

The GRU NN is obtained through a simplified LSTM NN. The LSTM NN has a complex gate structure, so detection with LSTM NN is a complex and time-consuming process [15]. Comparatively, the simplified version of LSTM NN, GRU NN, has condensed the gate structure into two, namely, reset gate and update gate. The reset gate can control the discarding of the previous-time state in the GRU cell structure, while the update gate can control the discarding of the previous-time memory cell information. Figure 4 shows the GRU structure.

The update gate of the *j*th GRU cell at time *t* can be calculated as follows:

(12) $$\begin{array}{c} Z_{t}^{j}=\sigma {\left(W_{z}x_{t}+U_{z}h_{t-1}\right)}^{j}. \end{array}$$

The reset gate at time *t* is calculated as follows:

(13) $$\begin{array}{c} r_{t}^{j}=\sigma {\left(W_{r}x_{t}+U_{r}h_{t-1}\right)}^{j}. \end{array}$$

Hence, equation (14) can be obtained.

(14) $$\begin{array}{c} h_{t}^{j}=\left(1-z_{t}^{j}\right)h_{t-1}^{j}+z_{t}^{j}\tanh\left(W_{r}x_{i}+U{\left(r_{r}⊙h_{t-1}\right)}^{j}\right.. \end{array}$$

### 3.2. Data Processing

#### 3.2.1. Data Processing Flow of Text Emotion Analysis Model

Here, the semantic characteristics of English texts are analyzed using the DL model. The specific process includes several steps: the collection of English text data, the preprocessing of English text data, the vectorization of the obtained data, and the establishment of the optimal DNN model and test. The collected English text semantic data are divided into two parts: a test set and a training set [16, 17].

#### 3.2.2. Data Preprocessing

**Figure 5:**
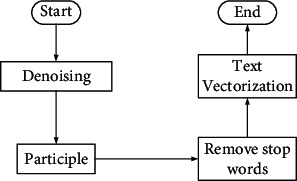
Preprocessing flowchart.

The data of the obtained English texts are preprocessed, just like all other experiments of semantic feature analysis. Preprocessing can filter out the irrelevant data to reduce the experimental error [18, 19]. The flowchart of preprocessing is shown in Figure 5.

#### 3.2.3. Improved SO-PMI (SO-Pointwise Mutual Information) Algorithm

The original point mutual information (PMI) algorithm is shown in the following equation:

(15) $$\begin{array}{c} \mathrm{PMI}\left(w_{1},w_{2}\right)=log2\frac{p\left(w_{1},w_{2}\right)}{p\left(w_{1}\right)p\left(w_{2}\right)}. \end{array}$$

The calculation of the SO-PMI algorithm is shown in the following equation:

(16) $$\begin{array}{c} \mathrm{SO}-\mathrm{PMI}\left(wor\;d\right)=\sum \mathrm{PMI}\left(wor\;d,Pwor\;d\right)-\sum \mathrm{PMI}\left(wor\;d,Nwor\;d\right). \end{array}$$

First, the SO-PMI distinguishes positive emotional English texts from negative emotional English texts, classifies and calculates the texts, and then subtracts them to obtain the emotional tendency of the English text [20]. However, the selection of different types of English texts requires manual intervention and high professionalism, so personnel selection is very meticulous. Moreover, every time a new English word appears, the previous English emotional dictionary should be adjusted, making the establishment of a reliable English emotional dictionary extremely difficult [21, 22]. Given these problems, the SO-PMI algorithm is specifically chosen. Equations (17) and (18) are used for PMI calculation:

(17) $$\begin{array}{c} \mathrm{PMI}\left(w,pos\right)=log\frac{p\left(pos|w\right)}{p\left(pos\right)}, \end{array}$$

(18) $$\begin{array}{c} \mathrm{PMI}\left(w,\mathrm{neg}\right)=log\frac{p\left(\mathrm{neg}|w\right)}{p\left(\mathrm{neg}\right)}, \end{array}$$

where *w* represents any word appearing in English text filtered out by the TF-IDF algorithm; *P* (POS) refers to the occurrence probability of positive emotional words in the English text dataset; *P*(POS*|w*) denotes the probability of word *w* appearing in the whole English text dataset [23]; PMI (*w*, POS) stands for the correlation between word *w* and positive emotional words in English text; and PMI (*w*, POS) > 0 indicates that the word *w* belongs to the positive emotional type, and the greater the value is, the higher the positive emotional intensity of word *w* is. On the contrary, the smaller the PMI (*w*, POS) is, the weaker the positive emotional intensity of word *w* is. PMI (neg) refers to the occurrence probability of negative emotional words in the English text dataset. *P*(neg*|w*) denotes the probability of word *w* appearing in the whole English text dataset. PMI (*w*, neg) stands for the correlation between word *w* and negative emotional words in English text. PMI (*w*, neg) > 0 indicates that the word *w* belongs to the negative emotional type, and the greater the value is, the higher the negative emotional intensity of word *w* is. On the contrary, the smaller the PMI (*w*, neg) is, the weaker the negative emotional intensity of word *w* is.

Next, the emotional tendency SO (*w*) of word *w* can be calculated by subtracting PMI (*w*, neg) and PMI (*w*, POS) according to the original SO-PMI algorithm:

(19) $$\begin{array}{c} \mathrm{SO}\left(w\right)=PMI\left(w,pos\right)-PMI\left(w,neg\right). \end{array}$$

When SO (*w*) > 0, the word *w* belongs to the positive emotional type, and the larger SO (*w*) is, the higher the positive emotional intensity of the word *w* is. When SO (*w*) gets closer to 0, the word *w* belongs to the neutral emotional words. When SO (*w*) < 0, the word *w* belongs to the negative emotional type, and the smaller SO (*w*) is, the higher the negative emotional intensity of word *w* is.

#### 3.2.4. Experimental Design

The efficiency of the improved SO-PMI algorithm is verified for English text feature analysis through the establishment of multiple models [24].

1. *CNN*. The CNN model only contains one convolution layer, and the feature analysis is completed through the combination of the convolution kernels. Totally, there are 120 convolution kernels in the convolution layer.
2. *LSTM NN*. algorithm is verified through the Bi-LSTM (bidirectional LSTM) NN, each layer of which contains 50 LSTM NN units.
3. *GRU NN*. The SO-PMI algorithm is verified through the dual-channel GRU NN model, each layer of which contains 50 GRU NN units.

**Figure 6:**
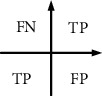
Confusion matrix.

English text feature classification can be detected through the following indexes: *F*1 score, recall, precision, and accuracy.  Figure 6 shows these indexes expressed by a confusion matrix.

**Figure 7:**
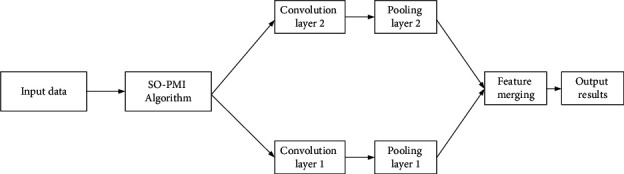
Flowchart of model operation.

In Figure 7, TP represents the number of positively predicted positive English texts, and FN represents the number of negatively predicted positive English texts. FP represents the number of positively predicted negative English texts, and TN represents the number of negatively predicted negative English texts.

The precision of the positive tendency (*P*+) and the negative tendency (*P*−) is calculated as in equations (20) and (21), respectively.

(20) $$\begin{array}{c} P+=\frac{\mathrm{TP}}{\mathrm{TP+FP}}, \end{array}$$

(21) $$\begin{array}{c} P-=\frac{\mathrm{TN}}{\mathrm{TN+FN}}. \end{array}$$

The recall rate of the positive tendency (*R*+) and the negative tendency (*R*−) is calculated as in equations (22) and (23), respectively.

(22) $$\begin{array}{c} R+=\frac{\mathrm{TP}}{\mathrm{TP+FN}}, \end{array}$$

(23) $$\begin{array}{c} R-=\frac{\mathrm{TP}}{\mathrm{TP+FN}}. \end{array}$$

The positive tendency (*F*1+) and the negative tendency (*F*1−) are calculated as in equations (24) and (25), respectively.

(24) $$\begin{array}{c} F1+=\frac{2\times \left(P+\right)\left(R+\right)}{\left(P+\right)+\left(R+\right)}, \end{array}$$

(25) $$\begin{array}{c} F1-=\frac{2\times \left(P-\right)\left(R-\right)}{\left(P-\right)+\left(R-\right)}. \end{array}$$

The accuracy of the whole model is calculated as follows:

(26) $$\begin{array}{c} \mathrm{accuracy=}\frac{\mathrm{TP}}{\mathrm{TP+FN+FP+TN}}. \end{array}$$

### 3.3. Construction of Dual-Channel CNN Model

The dual-channel CNN model is featured by two convolution channels, both of which correspond to the pooling layer and the convolution layer, respectively. Therefore, interference will not occur in the pooling and convolution calculations [25], and the two CNN can train and construct the whole model with the text data simultaneously, without mutual influence. The emotional attributes of short text can be minimized, and then the functions extracted from these two channels are folded and input into the classifier that determines the emotion of the text [26, 27]. Each channel of the CNN directly affects the original data, and then the subsequent layers of the multilayer CNN will affect the processed data, so the CNN can extract more direct functions from these two channels [28]. Figure 7 shows the operation process of the model.

#### 3.3.1. Data Input

The obtained English text data are input into the dual-channel CNN model through the feature matrix. Different from the single-channel CNN, the dual-channel CNN model inputs the word vectors of English text into different channels according to their features.

#### 3.3.2. Convolution Layer and Pooling Layer

The convolution layer of the dual-channel CNN operates just the same as that of single-channel CNN, which extracts text features from English text data with the convolution kernel [29].

#### 3.3.3. Feature Merging

Dual-channel CNN has two independent convolution channels, as well as a merging layer. The merging layer can extract the features of two independent convolution channels to establish a complete feature matrix that is input into the network to complete feature analysis of English text.

#### 3.3.4. Emotion Classification Using the Softmax Classifier

After the above steps 1, 2, and 3, the feature analysis and calculation of English text are realized through the softmax classifier [30].

### 3.4. Improved Dual-Channel CNN Model

#### 3.4.1. Dual-Channel CNN + Bi-LSTM

CNN can extract local features from English text big data. CNN is less sensitive to the temporal characteristics of the text. To better combine the advantages of CNN and LSTM, a CNN model combined with LSTM NN is proposed, namely, the dual-channel CNN + Bi-LSTM model. Specifically, the LSTM NN is connected with the whole model, and the experimental results are verified. Firstly, the data are input into the LSTM NN to get the synchronization information, and then they are input into the fully connected layer for subsequent operation. Here, the result of a common LSTM dual-channel CNN is compared with the proposed CNN + Bi-LSTM model, and the performance of the dual-channel CNN and the ordinary LSTM NN is compared.

#### 3.4.2. Dual-Channel CNN + Bi-GRU (Bidirectional GRU)

After simplification of the LSTM NN, GRU NN is obtained. Based on the above model, a Bi-GRU NN is used to replace the Bi-LSTM NN in the CNN model. Then, the dual-channel CNN + Bi-LSTM and CNN + Bi-GRU are compared and analyzed.

#### 3.4.3. Dual-Channel CNN with Attention Mechanism

The attention mechanism imitates the human visual mechanism. When people recognize a scene through vision, they do not look at all the details but rather focus on the key points of the whole scene. Key points are more helpful to understand the scene. When the weights are the same, the performance of the proposed model for English text feature analysis will decline, where the attention mechanism comes to play and further improves the performance of the proposed model for English text feature analysis.

#### 3.4.4. Comparative Experiment Design

To enhance the data contrast of this experiment, the DL algorithm and SVM (support vector machine) algorithm are used for the comparative analysis between the models to further verify the effectiveness of the attention mechanism model + GRU NN + dual-channel NN in text feature analysis.

## 4. Analysis of Experimental Results

### 4.1. Vector Training Model of Emotional Words

**Figure 8:**
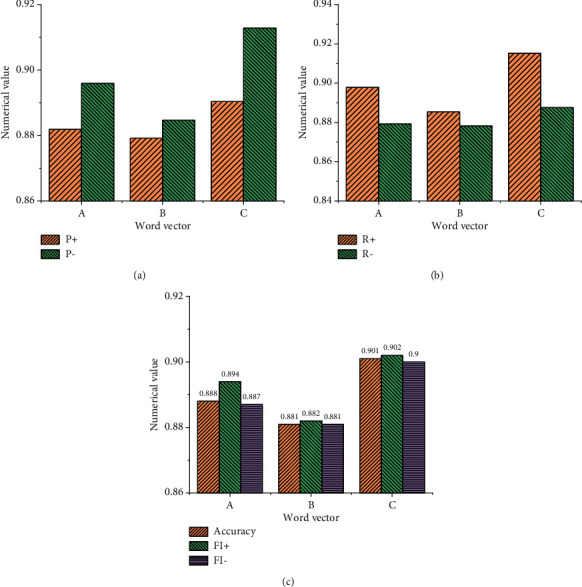
Statistical results of emotional word vector training model. (a) Ordinary English word vector. (b) The vector of English emotional words without word frequency. (c) English emotional word vector with word frequency.

Figure 8 shows the model training results using the emotional word vector.

**Figure 9:**
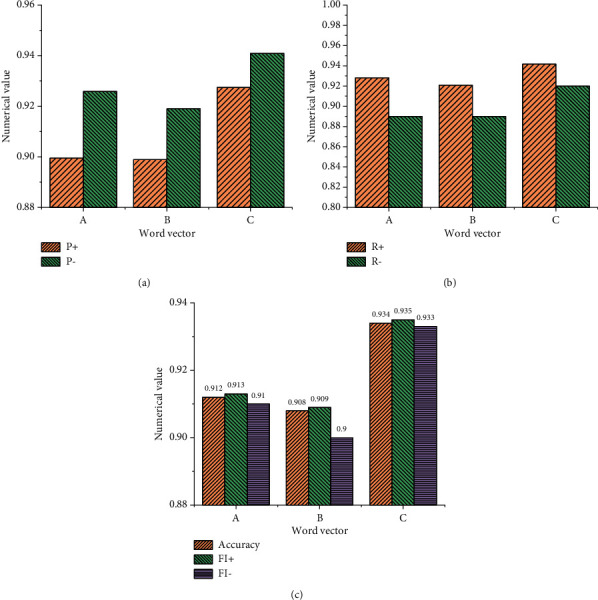
Statistical results of the LSTM NN training model for emotional word vector. (a) Common English word vector. (b) The vector of English emotional words without word frequency. (c) English emotional word vector with word frequency.

**Figure 10:**
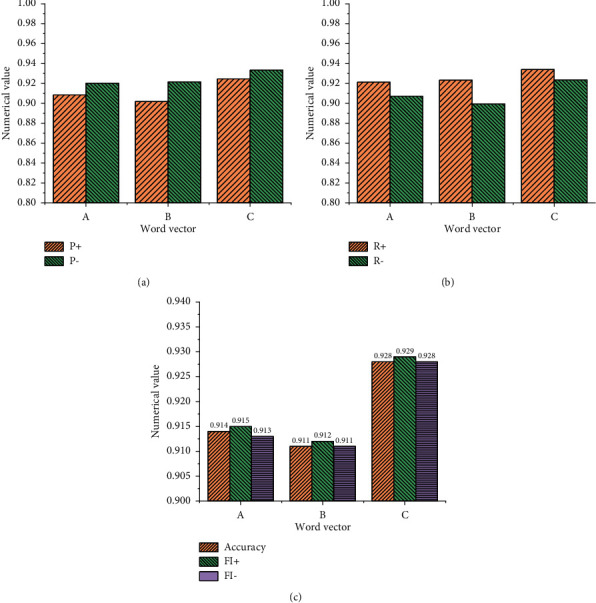
Statistical results of the GRU NN training model for emotional word vector. (a) Common English word vector. (b) The vector of English emotional words without word frequency. (c) English emotional word vector with word frequency.

Figure 9 implies that when the word frequency is not added to the training model, the result of the emotional word vector obtained by the model is relatively poor, and the parameters of the model have not improved but have decreased. Moreover, the accuracy of the model without word frequency is lower than that of the ordinary model by about 0.68%. This experiment also shows that the detection quality is relatively low when the word frequency is not added to the model, and the emotional word vector based on this model does not perform well in the emotional feature analysis of English text. Figure 9 shows the statistical results using the LSTM NN model, and Figure 10 demonstrates the statistical results using the GRU NN model.

Figures 9 and 10 suggest that the results of the LSTM NN model and GRU NN model are consistent when they use different types of English word vectors. Data comparison shows that when English word frequency is not added in the GRU NN, the accuracy of the model declines not obviously. Meanwhile, the comparative analysis indicates that when the English word frequency vector is not added into the model and only the common word vector is used in the model, the difference between the two is not big, and the accuracy of the word vector training model decreases slightly. However, when English word frequency is added into the model, the accuracy of the model will be significantly improved, especially, the LSTM NN model. Therefore, the comparative analysis of the three groups of experiments proves that the improved SO-PMI algorithm can automatically establish the English emotion dictionary, and the method of adding the semantic feature information from the English emotion dictionary to the word vector can be realized.

### 4.2. Comparative Analysis Experiment under Dual-Channel CNN

**Figure 11:**
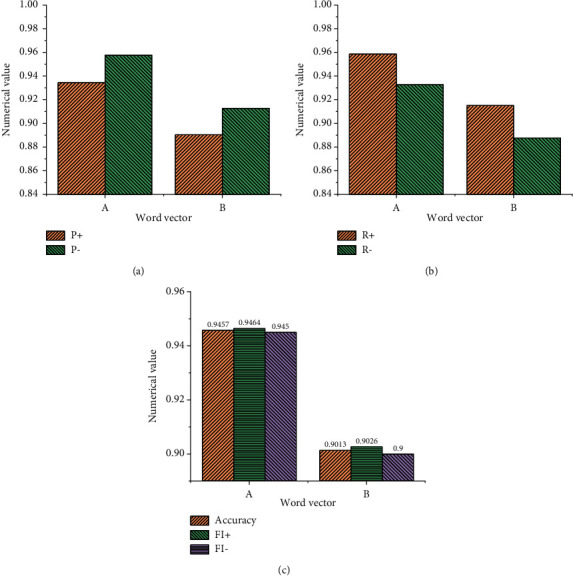
Comparison of single-channel CNN model and dual-channel CNN model results. (a) Single-channel CNN model. (b) Dual-channel CNN model. (c) Comparison of accuracy difference between dual channel CNN and single channel CNN.

Figure 11 shows the comparison of the results of single-channel and dual-channel CNN models.

**Figure 12:**
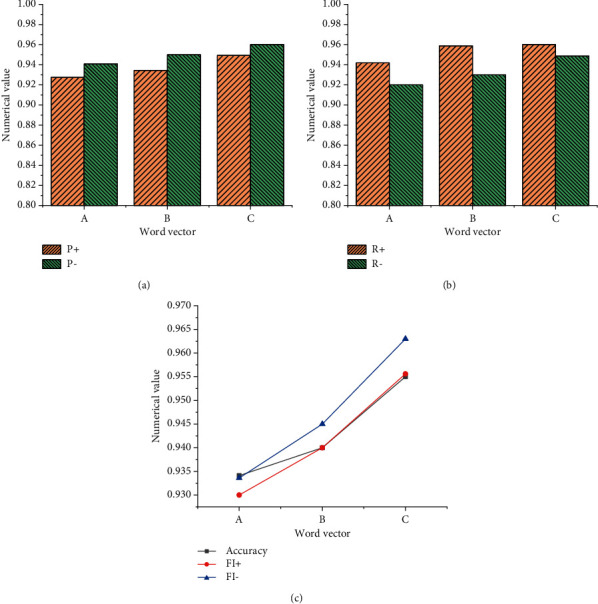
Statistical comparison of the experimental results of dual-channel CNN and LSTM NN. (a) LSTM NN. (b) Dual-channel CNN. (c) LSTM NN + dual-channel CNN.

Figure 11 implies that there is a big difference between the accuracy of the dual-channel CNN and the single-channel CNN in the text dataset. Comparatively, the accuracy of the English text dataset established by the dual-channel CNN can reach 96%, which is 4.45% higher than the single-channel CNN model. The results also show that the model established by the dual-channel CNN can extract more comprehensive semantic features of big data English text. Compared with the single-channel CNN, the dual-channel CNN model shows better utilization value in the short text of e-commerce reviews, At the same time, the effect of semantic feature analysis of English text is very significant.  Figure 12 shows the comparison of the experimental results between the dual-channel CNN and LSTM NN.

**Figure 13:**
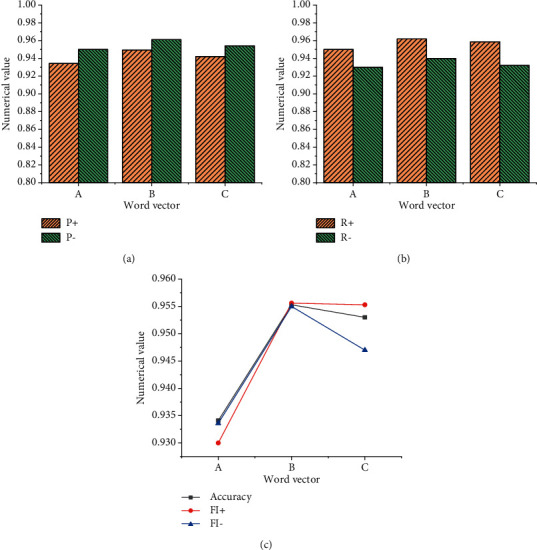
Comparison of experimental results. (a) Dual-channel CNN. (b) Dual-channel CNN + LSTM NN. (c) GRU NN + dual-channel CNN.

Figure 12 shows that the semantic feature analysis effect of the LSTM NN model in the English text dataset is not as good as that of the dual-channel CNN model. The comparative analysis of the data between dual-channel CNN and LSTM NN proves that when LSTM NN is added, the model performance in the semantic feature analysis of English text is improved, from 94.32% to 95.41% compared with that without LSTM NN. Hence, the model performance on time sequence feature analysis has improved when the LSTM NN is added, so that the performance of the dual-channel CNN model in English text features is further improved.  Figure 13 shows the comparison of experimental results among dual-channel CNN, dual-channel CNN + LSTM, and GRU NN.

**Figure 14:**
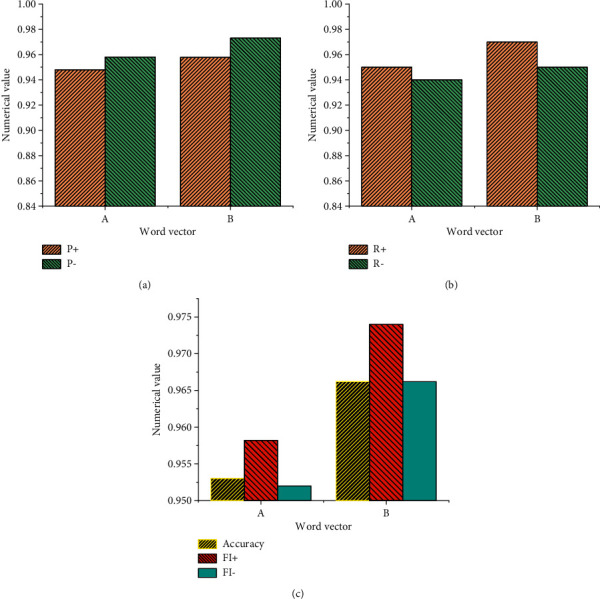
Experimental results. (a) GRU NN + dual-channel CNN with attention mechanism. (b) GRU NN + dual-channel CNN without attention mechanism. (c) Accuracy comparison of semantic feature analysis.

Under the comparative analysis of the GRU NN + dual-channel CNN model and the dual-channel CNN + LSTM NN model, the GRU NN + dual-channel CNN model is better for the detection of English text semantic features, but the performance increase is less obvious from the dual-channel CNN model to GRU NN + dual-channel CNN. After respective model training, the semantic feature analysis time of the dual-channel CNN + LSTM NN model is longer. Here, the GRU NN + dual-channel CNN model is selected for the experiment because it ensures a higher accuracy of semantic feature analysis, improves the analysis speed of the model, and has very good practical value.  Figure 14 shows the experimental detection results of GRU NN + dual-channel CNN after the attention mechanism is added.

Figure 14 reveals that when attention mechanism is added, the accuracy of semantic feature analysis of GRU NN + dual-channel CNN model is improved by nearly 1.3%. Thus, the expected results of adding attention mechanism are well obtained, which improves the accuracy of the GRU NN + dual-channel CNN model. Obviously, it is a good method to apply the attention mechanism to GRU NN + dual-channel CNN model.

## 5. Conclusion

Here, the application of the dual-channel CNN algorithm is mainly studied for the semantic feature analysis of English text big data. First, LSTM NN and GRU NN are introduced, and their effects on feature analysis of English text data are analyzed. Then, the improved SO-PMI algorithm is used to analyze the semantic features of English text big data. Finally, a dual-channel CNN model is implemented. Through experiments, it is found that the effect of dual channel CNN model in English text semantic feature analysis is significantly different before and after adding LSTM-NN, and the effect in semantic feature analysis is improved from 94.32% to 95.41%. LSTM-NN model can improve the ability of time feature analysis of the model, so as to further enhance the ability of dual channel CNN model to analyze English text features. Meanwhile, the GRU NN model has a better detection effect than the LSTM NN, but the performance increase from the dual-channel CNN model to GRU NN + dual-channel model is less obvious. The model training experiment shows that the LSTM NN + dual-channel CNN model takes more time in semantic feature analysis than the GRU NN + dual-channel CNN model. When the attention mechanism is added, the accuracy of semantic feature analysis of the GRU NN + dual-channel CNN model is improved by nearly 1.3%, and the expected results of adding attention mechanism are well obtained, which improves the accuracy of the GRU NN model. Therefore, GRU NN + dual-channel CNN + attention mechanism model is more suitable for semantic feature analysis in English text big data. However, there are still some limitations: the experiment has not involved specific analysis of different types of English parts of speech, such as adjectives, nouns, and verbs, so the addition of parts of speech analysis should be considered in the follow-up research to further improve the semantic feature analysis ability of the model.

## Data Availability

The data used to support the findings of this study are available from the corresponding author upon request.
